# Knowledge, Attitudes, and Practices of Consumers on Food Allergy and Food Allergen Labeling: A Case of Lebanon

**DOI:** 10.3390/foods12050933

**Published:** 2023-02-22

**Authors:** Hanin Chafei, Marwa Diab El Harake, Imad Toufeili, Samer A. Kharroubi

**Affiliations:** Department of Nutrition and Food Sciences, Faculty of Agricultural and Food Sciences, American University of Beirut, Riad El Solh, P.O. Box 11-0236, Beirut 1107-2020, Lebanon

**Keywords:** food allergy, food labels, precautionary advisory labeling, knowledge, attitudes, practices, purchasing habits, Lebanon

## Abstract

The epidemiology of food allergies is increasing worldwide. International labeling standards were developed to enhance consumers’ awareness of allergen-free foods. The main objective of the present study is to assess the characteristics of allergen labeling and consumers’ knowledge, attitudes, and purchasing habits of food products with allergens in Lebanon. We evaluated the allergen labeling of 1000 food products form Lebanese supermarkets. A random sample of 541 consumers was recruited through an online survey (November 2020–February 2021). Descriptives and regression analysis were conducted. Results showed that wheat represents the largest group of food allergens on food labels, followed by milk and soybean. Furthermore, 42.9% of supermarket food products had a precautionary allergen labeling with “may contain traces of allergens”. The majority of food products complied with local regulations for locally manufactured and imported products. One-quarter of survey respondents had a food allergy or were caregivers of food-allergic individuals. Regression analyses showed that “previous experience of a severe reaction” was negatively associated with food allergy-related knowledge and attitude scores respectively (*β* = −1.394, 95% CI: (−1.827, −1.034) and *β* = −1.432, 95% CI: (−2.798, −0.067)). The findings of this study provide practical insights on food allergy labeling issues for stakeholders and policymakers in the food supply chain.

## 1. Introduction

Food allergy (FA) is a growing public health concern. FAs are serious and potentially life-threatening medical conditions which can lead to health issues, including severe reactions, such as anaphylaxis and reduced quality of life [1,2]. The Centers for Disease Control and Prevention (CDC) reported that FAs affect 15 million people, responsible for approximately 30,000 emergency department visits, and cause 150 to 200 deaths every year in the United States [3]. However, preventing and/or mitigating the severe health consequences of FA can be accomplished by strict avoidance of food allergens and adhering to relevant labeling on foods.

The global prevalence of FAs has been reported to range between 1% and 10% and is estimated to be around 2.5% according to the World Allergy Organization [1,2]. In the US, for example, FA affects an estimated 5% of adults and 8% of children, with more than one food allergy reported in 2.4 percent of all children, or about one-third of the children with a reported food allergy [4]. According to the World Allergy Organization, the prevalence rates of FA in children were highest in Finland, Poland, and the UAE and lowest in Thailand, Brazil, and Turkey [5]. However, data on FA prevalence rates in the Middle East and North Africa (MENA) region are rather scarce, and difficult to locate. In a recent study from Saudi Arabia, the prevalence of FA was estimated to be around 19.7% [6]. In Lebanon, the prevalence of FA was estimated to be 3.2% in adults and 4.1% in infants [7], and in 2022, 6% of the children were reported to have FA [8].

To protect food-allergic consumers from accidental exposure to culprit foods, several countries have developed allergen labeling regulations and guidelines. However, these regulations differ markedly around the world. In well-developed countries, food allergen labeling requirements are based on the prevalence of allergens and scientific research. Other countries adopt one or several regulations (fully or partially) including those of the US Food and Drug Administration, European Union (EU), and the Codex Alimentarius. In Lebanon, it is mandatory to list 13 major food allergens that cause allergies and intolerances on the labels of prepackaged and non-packaged foods. These include sesame seed, mustard, lupin, milk, soya, fish, egg, crustacean, peanut, nut, wheat, sulfite, and celery (NL 206, 2017) [9]. However, potential contamination of food with allergen residues can happen at various points across the food supply chain including the common food industry malpractice of using the same equipment to produce allergenic and non-allergenic food products. Such malpractice can result in the presence of detectable residues in foods and may pose health risks to people suffering from food allergies. Consequently, food manufacturers started to use precautionary advisory labeling on packaged food to alert consumers to any possible risk of allergen exposure from these foods. Various types of advisory labeling are used worldwide such as “may contain x” and “manufactured in a shared facility with x” [10]. Several studies have been carried out to assess the compliance of packaged food products with these regulations [11,12]. Other studies focused on the impact of these practices on FA sufferers [13,14]. Yet, unfortunately, there are still gaps in food allergen labeling knowledge and practices among consumers.

In the Middle East, the majority of studies have examined food safety knowledge, attitudes, and practices (KAP) of food handlers in food service establishments (FSEs) in Lebanon, Turkey, Jordan, UAE, Qatar, Saudi Arabia, Palestine, and Egypt [15,16,17,18]. In most of these studies, the food safety knowledge was inadequate due to a combination of a low proportion of skilled food handlers and limited access to adequate food safety education. However, few studies in the region examined KAP related to FA. In Saudi Arabia, one study showed that adherence to the newly enacted Saudi Food and Drug Authority (SFDA) allergen-labeling legislation was low among Saudi consumers and there was a need to increase the level of awareness among adults suffering from FA [19]. In Kuwait, the prevalence of perceived FA was low and experienced mostly in early childhood in a poll of Kuwaiti students attending Kuwait University [20].

In Lebanon, two studies examined KAP towards food allergens and allergies, in addition to food allergen labeling [21,22]. Another recent study explored KAP related to FA of food service workers and managers at Lebanese restaurants and underscored the need for FSEs, including mainly restaurants, to be proactive by training their staff on FA and implementing effective food allergy management plans [23]. Given the dearth of information on the contribution of food labeling to the management of FAs, the objectives of the present study are to: (i) assess the characteristics of FA labeling on packaged foods; (ii) verify the compliance of food labels according to Lebanese requirements for mandatory labeling of foods that contain potential allergens; (iii) explore KAPs of consumers toward allergen labeling, and; (iv) investigate the sociodemographic determinants of KAP related to allergen labeling among the study participants. Apart from their contribution to the body of knowledge and raising awareness on food allergies, findings from the present study are crucial for the public health sector in Lebanon and the MENA region.

There are two important issues in which the present study may be useful for Lebanon. First, all food manufacturers are required to include the list of ingredients in the labeling of prepackaged foods. However, the government does not mandate the presence of precautionary labeling [24]. No recent studies have examined the characteristics of FA labeling of packaged foods and/or assessed the compliance of food labels according to the Lebanese requirements for mandatory labeling of foods that contain potential allergens. A second key challenging issue is related to the deteriorating socio-economic situation in Lebanon. The financial and economic crises in Lebanon, overlapped with the COVID-19 pandemic, all weighed down heavily on food systems, which threatened to exacerbate food insecurity [25,26,27]. As a result, this led to a significant decrease in food supply and rise in food prices. This in turn put food-allergic patients under stress and at higher risk of accidental exposure to food allergens.

## 2. Materials and Methods

### 2.1. Study Setting and Population

Market analysis of packaged food products was conducted at two major supermarket chains in Lebanon between November 2020 and January 2021. The supermarkets were chosen based on the information available online and the suggestions of local citizens. One multinational and one local supermarket chain frequented by consumers with different socioeconomic statuses were included in the survey.

During the market analysis, products were chosen randomly from different manufacturers. For each product, digital photographs were taken, and all images were verified twice to link the match between the label captions and products. The information extracted from the images consisted of the following: product and brand names, country of origin, list of ingredients, special emphasis on known allergens and how such emphasis was presented, and any advisory statements. In addition, the possibility of the warnings being in a covert place, removable by sealing, or difficult to visualize was checked, as well as the possibility of incorrect spelling of the allergens and the claim about the absence of allergens. Both local and imported packaged food products were verified for their compliance with local regulations for the characteristics of allergen labeling regulations (General Standard for the Labeling of Prepackaged Food, NL 206). For products with multiple package sizes, only one size was included in the analysis to avoid bias. Moreover, duplicate products found in the two supermarkets were recorded only once.

### 2.2. Data Collection and Survey

A descriptive cross-sectional study was conducted online among Lebanese citizens or residents of Lebanon that are at least 18 years of age and those who usually participate in grocery shopping in their households to examine their purchasing habits, attitudes, and use of food allergen labeling. Sample size calculations showed that a minimum of 577 participants ought to be recruited to estimate a prevalence of 50% with a 95% CI and a margin of error of 5% and a design effect of 1.5. The sample size was calculated using the World Health Organization (WHO) sample size calculator Available online: https://cdn.who.int/media/docs/default-source/ncds/ncd-surveillance/steps/sample-size-calculator.xls?sfvrsn=ee1f4ae8_2 (accessed on 11 October 2020) [28]. Assuming a response rate of 0.8, a representative sample of 720 participants was selected.

The research team was CITI-certified and received training on conducting research with human subjects according to AUB Institutional Review Board (IRB) regulations before the initiation of the study. Data collection took place through an online survey that was posted on social media platforms (WhatsApp groups, Facebook pages, Instagram, and Twitter) between January 2021 and March 2021. Before starting the survey, participants were asked to complete a consent form that appeared on their screen. The survey was based on previous similar studies [13,14] and divided into three sections. The first section included questions related to participants’ sociodemographic characteristics such as age, gender, educational level, and income. The second section included questions related to their purchasing habits, including the amount of grocery shopping per month and the frequency of label use while purchasing. This was followed by a section that included questions related to the knowledge, attitude, and use of allergen labeling. The completion of the survey should take approximately 5–10 min. Participants’ identity was completely anonymous and participation in the survey was voluntary.

### 2.3. Statistical Analysis

Data were extracted, cleaned, entered, and statistically analyzed using the Statistical Package for the Social Sciences (SPSS) version 24.0. Knowledge scores were calculated for each participant by adding the number of correct answers (out of 5). Scores for knowledge and best practice were calculated using by adding the scores of the different items (statements) and transforming into scores over 100. For the attitude scores, point values to each response were assigned as follows: strongly disagree = 1, disagree = 2, unsure = 3, agree = 4, and strongly agree = 5. Then, we computed each participant’s average response to the 5 attitude questions by summing up positive attitudes.

Descriptive statistics were presented as means and standard deviations (SD) for continuous variables or as frequencies and proportions for categorical variables. Pearson’s correlation was used to correlate knowledge scores with sociodemographic characteristics. Chi-square tests were conducted to compare the types of emphasis used when declaring allergens on the ingredient list between local and imported food products. Univariate and multivariate linear regressions were applied to determine which factors were associated with the knowledge scores and attitude scores. In the regression models, knowledge and attitude scores were used as the dependent variables, with sociodemographic factors as independent variables. Characteristics that showed statistical significance in the univariate analysis were included in the multivariate model as independent variables. Results from the linear regression models were expressed as Beta coefficients (*β*) with 95% confidence intervals (CI). The beta coefficients from the univariate and multivariate linear regression models were estimated using Ordinary Least Squares. For all analysis conducted, a *p*-value of less than 0.05 was considered statistically significant.

## 3. Results

### 3.1. Market Analysis

#### 3.1.1. Samples

Overall, 1000 food product labels were analyzed. Of these, 951 products had allergen labeling and/or precautionary allergen labeling. The remaining 49 products had no labeling, given these products were naturally allergen-free products or did not contain allergens in their ingredient lists. Thus, the alerts presented in the other samples were analyzed.

#### 3.1.2. Categories

The analyzed foods were divided into 14 categories: bakery, baby food, chilled food, frozen food, jam and spreads, ready meals, beverages, canned food, sauces and dressings, dessert mixes, instant food, salty snacks, chocolate, and biscuits. Results revealed that bakery, and biscuits, followed by instant food (15%, 14%, and 13%, respectively), represented the highest amount of food analyzed, while baby food, beverages, and jams and spreads represented the lowest (4%, 3%, and 2%). The variation in the analyzed categories of food was due to the number of food products present at the time of the visit.

Among products which had precautionary allergen labeling, we analyzed frequency and type of advisory statement. Out of the 951 samples analyzed, 408 samples (42.9%) made use of precautionary allergen labeling. Among these, nine different precautionary statements were identified. The most frequently used precautionary statement was “may contain traces of x” (59.6%), followed by “may contain x” (27.7%), and “our facility handles” (4.4%).

It is perhaps worth mentioning that most of the products surveyed complied with the allergen labeling requirements of the Lebanese standard institution-LIBNOR (General Standard for Labeling of Prepackaged Foods) (94.1% for locally manufactured and 97.6% for imported products).

#### 3.1.3. Declaration of Allergens

Among the 951 products with allergen description, 908 products had a description of allergens in the ingredient list and 408 products had a description in the precautionary statement (Table 1). Of these, 365 products were found to have a description in both the ingredient list and precautionary statement.

#### 3.1.4. Special Declaration of Allergens

Recall that Chi-square tests were conducted to compare the types of emphasis used when declaring allergens on the ingredient list between local and imported food products. Results from Table 2 reveal that most of the imported products carried a special emphasis (bold) on the allergens presented on the labeling (60.5%). However, for the local products, more than half (51.7%) did not show special emphasis. Although the styles for special allergen declaration vary significantly (χ^2^ = 243.005, *p* = 0.001), a “Contains statement” was noted on most of the locally manufactured products (Table 2).

#### 3.1.5. Ambiguous Declaration

There were 72 instances of ambiguous labeling found in the food products where sources of ingredients were unknown. These can be divided into the types of flour (72.4%), (17.2%) spices, vegetable oil (5.2%), type of emulsifier used (3.4%), and source of lecithin (1.7%). Further, a total of 27 discrepancies (difference between ingredient list and contains statement) were found. In twelve food products, food allergens were listed in the ingredients but not included in the contain statement. For example, mustard was present in the ingredient list of a product but only declared “contains egg”.

In addition, eleven products labeled the allergens in the ingredient list, but also declared them in the precautionary statements. A frozen ready pizza meal declared milk and gluten in the ingredient list but also declared the following “produced in a factory that contains milk and gluten”. Moreover, there were two products with two advisory labels, a “may contain” statement and “produced in a factory that also handles”. Furthermore, there were two products where the allergen was present in French but was not translated to Arabic and English. However, the type of tree nut was not disclosed for 48.9% of products with advisory labels for tree nuts.

### 3.2. Survey Analysis

#### 3.2.1. Demographic Characteristics

Out of the 720 subjects sent of the questionnaire, 640 agreed to participate in this study, resulting in an 89% response rate. Of these, data were missing for 99 respondents, leaving 541 responses for analysis. Characteristics of the study population are presented in Table 3. The mean (SD) age of participants was 25.71 ± 7.65 years with majority being females as compared to males (81.8% vs. 18.3%). The majority of survey respondents were Lebanese (86.3%) and single (81.1%). As for the participants’ highest education level, the vast majority of the respondents had university education (87.6%). Further, approximately one-third of the participants resided in the South (31.2%), followed by Mount Lebanon (30.5%) and then Beirut (27.2%), whilst the remaining were in Beqaa (5.9%) and North (5.2%). Regarding current employment status, more than a third of participants (36.0%) were studying, whilst 44.0% of them were employed (full-time (33.8%) and part-time (10.2%)), and 19.8% were either seeking employment or unemployed. More than a third of participants (36.2%) had a total monthly income between LBP 1,000,000 and LBP 3,000,000, 26.1% of more than LBP 5,000,000, 25.5% between LBP 3,000,0000 and 5,000,000, and 12.2% had a monthly income below LBP 1,000,000. It is perhaps worth noting that, among food-allergic respondents, only 15.3% reported a history of severe allergic reaction (Table 3).

#### 3.2.2. Purchasing Habits of Participants in Relation to Allergen Labeling

Findings of purchasing habits for participants in relation to allergens are shown in Table 4. Results revealed that a majority of the study respondents (81.6%) always check the ingredient lists before purchasing a food item. However, slightly more than half of study respondents (54.7%) always check advisory statements before purchasing. Concerning the advisory labels, 17.5% always buy products labeled as “may contain allergens”. This percentage increased to 41.6% for those who buy products labeled as “may contain traces of allergens”. However, 59.1% and 34.3% always buy products labeled as “manufactured in a facility that also processes allergens” and “allergen-free”, respectively (Table 4).

About 58.5% experienced an accidental exposure, 26.3% were linked to failure in reading a food label, and 16.1% reported ignoring a precautionary statement and inappropriate labeling. However, the majority of food-allergic respondents and their carers suggested a bigger font, content amount of the allergen, and attractive and colorful symbols in order to separate allergen information from nutrition information to be added to the labels (68.6%).

#### 3.2.3. Food Allergen Labeling Knowledge Score

The mean (SD) food allergy knowledge score was 2.16 ± 0.98, with 14.6% of respondents were not aware that names of major allergens were required legally to be reported on labels. Further, 77.4% of respondents incorrectly believed that precautionary statements are required by law, and 17.5% reported that they did not know. A percentage of 42.3% of respondents believed that precautionary statements/advisory labels were based on the content amount of allergen present (Table 5).

Results from simple linear regression indicated that previous experience of a severe food allergic reaction and the governorate where the participants live were the main predictors associated with knowledge scores. More specifically, living in the North area was significantly associated with lower knowledge score as compared to Beirut area (*β* = −1.023, 95% CI: (−1.678, −0.367), *p* = 0.02). In addition, “previous experience of a severe reaction” was negatively associated with food allergen knowledge score (*β* = −1.43, 95% CI: (−1.827, −1.034), *p* < 0.001) (Table 6).

#### 3.2.4. Food Allergen Labeling Attitude Scores

Food allergen labeling attitude scores are presented in Table 7. Results reveal that more than half of the respondents (53.3%) disagreed or strongly disagreed that precautionary statements are easy to understand and considered helpful. About 70.8% and 78.1% also agreed or strongly agreed that E-numbers are provided, with no details and lack of information (i.e., manufacturer’s details) (Table 7). However, the majority (89.1%) agreed or strongly agreed on the usage of generic terms in the ingredients list (i.e., spices, flour).

Simple linear regression analysis showed that nearly all the respondents scored high attitude points. Few predictors such as employment status and previous experience of severe food allergic reaction were associated with attitude score. However, multiple regression analysis showed that “previous experience of a severe reaction” was negatively associated with food allergen attitude score (*β* = −1.432, 95%CI: (−2.798, −0.067), *p* = 0.04) (Table 8).

## 4. Discussion

The present study revealed novel findings in terms of assessing the characteristics of FA labeling on packaged foods and verifying the compliance of food labels according to the Lebanese requirements for mandatory labeling of foods that contain potential allergens. Furthermore, the study examined KAPs towards food allergen labeling in addition to their sociodemographic determinants among the study participants and demonstrated that those who have not experienced a serious FA reaction decreased FA-related knowledge and attitude scores.

Findings from the present study showed that the most declared allergens of foods in Lebanon include wheat (64%) followed by milk (50.9%) and soybean (29.3%). These are the same as the allergens indicated on food and beverages labels in Malaysia [29]. Likewise, a study conducted in Malawi reported that wheat was also the most frequently declared allergen (95%) followed by milk (64%) and soy (55%) [11]. In an extensive study surveying 10,254 packaged food products in six Latin American countries, the most commonly declared allergens were milk, including its derivatives (n = 3397; 52.32%), wheat and other cereals containing gluten (n = 3335; 51.36%), and soybean, including its derivatives (n = 2696; 41.52%) [12].

The presence of precautionary advisory labeling (PAL) on packaged food is intended to alert consumers to any possible risk of allergen exposure from these foods. However, these statements are not regulated in most countries. In the present study, 42.9% of the products carried precautionary statements, with the most commonly used statement being “may contain traces of x” (59.6%), followed by “may contain x” (27.7%), and “our facility handles” (4.4%). Similar results were reported in Latin America (33.2%) [12] and Serbia (33.9%) [30], where products had precautionary statements for one or more allergens; the most common type of PAL on pre-packaged foods analyzed in Serbia was “may contain traces of x (allergen)” (52.7%) [30], with a higher percentage being reported in Malaysia (94%) [29].

The findings from the present work further suggest that the prevalence of self-reported food allergies in this sample was relatively high (25.3%) when compared to adults in Saudi Arabia (21.4%) [31], and university students in the United Arab Emirates (8%) [32] and Kuwait (12%) [20]. Given that PAL are usually confusing to food-allergic consumers, many food-allergic consumers tend to ignore them [33], as reflected by 17.5% of participants always buying products labeled as “may contain allergens”. This proportion of consumers increased to 41.6% for those who buy products labeled as “may contain traces of allergens” and to 59.1% who always buy products labeled as “manufactured in a facility that also processes allergens”. Similarly, in the US and Canada, a study reported that 11% of respondents purchased food with “may contain” labeling, and 40% purchased food “manufactured in a facility that also processes allergens” [14]. In contrast, notably lower figures at 2.9% of food shoppers purchasing foods that bear “may contain x (allergen)” were reported from Serbia [30].

Notwithstanding cultural variations amongst different countries, the divergent and varied effects of the PAL statements on the purchasing practices of food-allergic consumers could be attributed, at least in part, to confusion in the minds of the consumers wrought by the use of different and seemingly complicated messages. This finding necessitates the development of straightforward and more nuanced statements to curtail the possibility of developing imprudent purchasing practices by food-allergic consumers.

Despite differences in the allergen-labeling regulations amongst different countries, the bulk of imported (97.6%) and locally manufactured food products (94.1%) complied with the national regulations. According to LIBNOR standards, the inclusion of a list of ingredients is mandatory (NL 206, 2017). This requirement may not be sufficient. For instance, it was reported that the use of special styles helps an allergic consumer to pay attention to allergen information on the label [34]. However, in the absence of a special emphasis regulating requirement, it is not surprising that almost half (51.7%) of all the surveyed locally manufactured products did not use any special emphasis when declaring allergens. Moreover, ambiguous labeling may confuse consumers as well. Label misinterpretation, the misunderstanding of non-specific terminology on food products, and a lack of PAL clarity were reported as the main causes of food allergies among consumers [33,35]. Additionally, the difference between the “contain” statement or allergy alert and the allergens declared in the ingredients list may be a disadvantage as consumers may rely only on the “contain” statement and ignore other allergens listed in the ingredients [36]. This might also explain the reason behind the high rate of accidental exposures (58.5%). Of these, 26.3% were linked to failure in reading a food label and 16.1% were related to ignoring a precautionary statement and inappropriate labeling. These results are similar to a Canadian study which recruited food-allergic individuals or their caregivers and found that, of 47.8% respondents who experienced an accidental exposure, 47.0% of the reported cases were attributed to inappropriate labeling, 28.6% were linked to failure in reading a food label, and 8.3% to ignoring a precautionary statement [36].

Furthermore, we identified important gaps in knowledge around allergen labeling and precautionary statements. For example, 77.4% of allergic individuals or caregivers of allergic individuals incorrectly believed that these statements were required by law. This finding is in alignment with a previous study reporting that almost half of the respondents in both the United States and Canada believed that these labels are requirement by law [14]. Results revealed that a previous serious allergic experience is significantly associated with higher knowledge and attitude scores. This might explain that food-allergic consumers seek more information in order to avoid serious illness.

With food labeling being the primary risk communication tool between the manufacturer and consumers, it is of great importance to understand the consumers’ reactions to such labels to ultimately reduce the FA risks. In general, studies showed that food-allergic consumers are not satisfied with the current labeling practices. Consumers preferred the use of symbols that indicate whether an allergen was present or not in the product along with a clearer and well-defined allergy content statement [23,37]. Moreover, consumers recommended improvements in font size, color, and shape of nutrition labels for clear and effective communication with allergic consumers [11,34,38]. In this study, food-allergic consumers and their caregivers agreed or strongly agreed that E-numbers and manufacturers’ details should be provided with more detail, which is consistent with a study conducted in Mauritius [13]. Additionally, they suggested a bigger font, content of the allergen, and attractive and colorful symbols to separate allergen information from nutrition information on the labels. In common with food-allergic consumers from other countries, most of the survey participants preferred the ingredients to be in bold and have a bigger font [23]. Having clearer, more specific, and consistent labeling, indicating a quantitative risk assessment (QRA), would improve purchasing decisions and communication around food allergen risk and safety among food allergy participants from Germany, Ireland, Netherlands, Spain, and the UK [39].

Limitations of the study should be noted. First, Lebanon is facing an economic crisis, so supermarkets visited were missing a lot of products. Hence, the surveyed products present at the time of data collection were limited. Additionally, the survey on knowledge of food allergen labeling was conducted online and participants were invited via social media which may lead to a selection bias. Further, all data were self-reported, so participants tend to overestimate their understanding and use of labeling, which is also subjected to information bias. Despite that, our study gave considerable insights to the food labeling practices and food-allergy-related knowledge, attitude, and practices of Lebanese consumers.

## 5. Conclusions

To the best of our knowledge, this study is the first to assess the characteristics of allergen labeling and consumers’ knowledge, attitudes, and purchasing habits of food products with allergens in Lebanon. The results showed that although the majority of the surveyed food products’ labeling declared the presence of food allergens according to the local regulations, allergic people are not very well-protected, since there are still multiple cases of missing, vague, and contradictory statements in the labels. Furthermore, our data also showed that there are gaps in knowledge and many misconceptions around precautionary allergen labeling that potentially affect the purchasing practices of food-allergic consumers. Consistent and clear labeling should increase consumers’ confidence while reducing accidental exposures. Thus, improved labeling rendering allergens better noticeable should contribute to more effective mitigation of FAs. Until such measures are in place, ongoing educational programs should be enhanced to better inform food-allergic patients and their caregivers about food-allergen labeling and how to purchase products. In addition, more work should be conducted to determine the levels of undeclared allergens, and evaluate the perceptions and frequency of food allergen labeling in food manufacturing companies.

The findings of our study are useful to policymakers, the food sector, and healthcare professionals to better manage food allergies in Lebanon. Identifying the allergens and food allergies in Lebanon will induce government decision makers to require allergen warning labels in the nation.

## Figures and Tables

**Table 1:** Prevalence of labeling for common food allergens in food products.

Allergen Category	Presence of Allergen in Ingredient List (*n* = 908) *n* (%)	Precautionary Statement (*n* = 408) *n* (%)
Wheat	581 (64)	90 (22.1)
Milk	462 (50.9)	106 (26)
Soya	266 (29.3)	166 (40.7)
Egg	119 (13.1)	135 (33.1)
Nut	90 (9.9)	229 (56.1)
Sesame	48 (5.3)	104 (25.5)
Mustard	29 (3.2)	43 (10.5)
Celery	22 (2.4)	35 (8.6)
Peanut	18 (2)	125 (30.6)
Fish	17 (1.9)	15 (3.7)
Sulfite	8 (0.9)	18 (4.4)
Crustacean	3 (0.3)	18 (4.4)
Lupin	3 (0.3)	15 (3.7)

**Table 2:** Types of emphasis used when declaring allergens on the ingredient list.

	Total	Local	Imported	Significance
Type of Emphasis	*n* (%)			
Bold	389 (42.7)	36 (11)	353 (60.5)	χ^2^ = 243.005 *p* = 0.001 *
Contains Statement	230 (25.3)	99 (30.3)	131 (22.5)	
Parenthesis	41 (4)	8 (2.4)	28 (4.8)	
Allergy Advice	27 (3)	13 (4)	14 (2.4)	
Enlargement Font	2 (0.2)	0	2 (0.4)	
Underlined	5 (0.5)	2 (0.6)	3 (0.5)	
Contrasting Color	1 (0.1)	0	1 (0.2)	
No Emphasis (Only present in the ingredient list)	220 (24.2)	169 (51.7)	51 (8.7)	

* indicates statistical significance at *p* < 0.05.

**Table 3:** Sociodemographic characteristics of the study population (*n* = 541).

	Total	Non-Food-Allergic (*n* = 404)	Food-Allergic
	(*n* = 541)		(*n* = 137)
Characteristics	*n* (%)		
Age	25.71 ± 7.65	25.78 ± 7.93	25.50 ± 6.79
Gender			
Male	99 (18.3)	71 (17.6)	28 (20.4)
Female	442 (81.8)	333 (82.4)	109 (79.6)
Marital Status			
Single	439 (81.1)	320 (79.2)	119 (86.9)
Married	102 (18.9)	84 (20.8)	18 (13.1)
Governorate			
Beirut	147 (27.2)	111 (27.5)	36 (26.3)
South	169 (31.2)	128 (31.7)	41 (29.9)
North	28 (5.2)	17 (4.2)	11 (8)
Mount Lebanon	165 (30.5)	126 (31.2)	39 (28.5)
Bekaa	32 (5.9)	22 (5.4)	10 (7.3)
Nationality			
Lebanese	467 (86.3)	346 (85.6)	121 (88.3)
Non-Lebanese	74 (13.7)	58 (14.4)	16 (11.7)
Educational Level			
Middle School	9 (1.7)	6 (1.5)	3 (2.2)
High School	53 (9.8)	41 (10.1)	12 (8.8)
University Degree (Bachelor)	285 (52.7)	215 (53.2)	70 (51.1)
University Degree (Masters/PhD)	189 (34.9)	138 (34.2)	51 (37.2)
Technical School	5 (0.9)	4 (1)	1 (7)
Current Employment Status			
Employed Full-Time	183 (33.8)	131 (32.4)	52 (38)
Employed Part-Time	55 (10.2)	42 (10.4)	13 (9.5)
Seeking Employment	69 (12.8)	47 (11.6)	22 (16.1)
Unemployed/Housewife	38 (7)	30 (7.4)	8 (5.8)
Retired	1 (2)	1 (2)	0
Student	195 (36)	153 (37.9)	42 (30.7)
Total Monthly Income			
<LBP 1,000,000	68 (12.2)	52 (12.8)	14 (10.2)
LBP 1,000,000–LBP 3,000,000	196 (36.2)	147 (36.4)	49 (35.8)
LBP 3,000,000–LBP 5,000,000	138 (25.5)	103 (25.5)	35 (25.5)
>LBP 5,000,000	141 (26.1)	102 (25.2)	39 (28.5)
Previous severe allergic reaction			
Yes			21 (15.3)
No			116 (84.7)

**Table 4:** Respondents’ purchasing based on food allergen labeling.

Variable	*n* (%)
Check the ingredient list present on label of packaged food before purchasing a food item	
Always	118 (86.1)
Sometimes	6 (4.4)
Purchasing the product for the first time	11 (8.0)
Never	2 (1.5)
Check the precautionary statements if present on label of packaged food before purchasing a food item	
Always	75 (54.7)
Sometimes	36 (26.3)
Never	26 (19)
Purchase Product with the Following Label	
“May Contain Allergens”	
Always	24 (17.5)
Sometimes	72 (52.6)
Never	41 (29.9)
“May Contain Traces of Allergens”	
Always	57 (41.6)
Sometimes	55 (40.1)
Never	25 (18.2)
“Manufactured in a Facility that also Processes Allergens”	
Always	81 (59.1)
Sometimes	38 (27.7)
Never	18 (13.1)
“Allergen-Free”	
Always	47 (34.3)
Sometimes	69 (50.4)
Never	21 (15.3)

**Table 5:** Respondents’ knowledge about food labeling laws.

Knowledge	*n* (%)
Food source names of major allergens required by law	
True	64 (46.7)
False	53 (38.7)
I don’t know	20 (14.6)
Advisory label required by law	
True	106 (77.4)
False	7 (5.1)
I don’t know	24 (17.5)
Advisory labels are based on amounts	
True	58 (42.3)
False	59 (43.1)
I don’t know	20 (14.6)

**Table 6:** Linear regression of food allergen knowledge scores with sociodemographic characteristics.

	Simple Linear Regression *β* Coefficient, (95% CI)	*p*-Value
Gender	0.214 (−0.201, 0.628)	0.310
Age	0.02 (−0.004, 0.045)	0.107
Marital Status		
Single (ref.)	0	
Married	0.014 (−0.482, 0.511)	0.954
Nationality (Lebanese)	−0.112 (−0.633, 0.41)	0.673
Governorate		
Beirut (ref.)	0	
South	−0.043 (−0.477, 0.392)	0.846
North	−1.023 (−1.678, −0.367)	0.002 *
Bekaa	0.150 (−0.530, 0.830)	0.663
Mount Lebanon	−0.237 −0.677, 0.202)	0.288
Educational Level		
Middle School (ref.)	0	
High School Diploma	−0.083 (−1.209, 1.043)	0.884
Undergraduate (Bachelor’s Degree)	−0.105 (−1.133, 0.924)	0.841
Master’s degree	0.843 (−0.193, 1.88)	0.11
Technical School	1.667 (−0.348, 3.681)	0.104
Employment Status		
Employed (Full Time) (ref.)	0	
Employed (Part Time)	0.288 (−0.313, 0.89)	0.344
Seeking employment	0.351 (0.142, 0.845)	0.161
Unemployed/Housewife	0.317 (0.419, 1.054)	0.396
Student	−0.201 (−0.603, 0.202)	0.326
Total Income		
<LBP 1,000,000 (ref.)	0	
LBP 1,000,000–LBP 3,000,000	0.149 (−0.439, 0.736)	0.617
LBP 3,000,000– LBP 5,000,000	−0.014 (−0.626, 0.598)	0.963
>LBP 5,000,000	−0.284 (−0.887, 0.319)	0.353
Previous experience of a severe reaction	−1.430 (−1.827, −1.034)	*p* < 0.001 *

* Estimates shown in bold are those that are statistically significant at *p* < 0.05.

**Table 7:** Attitudes toward food allergen labeling.

Attitudes	*n* (%)
Precautionary statements are easy to understand and considered helpful	
Strongly Agree	13 (9.5)
Agree	10 (7.3)
Neutral	41 (29.9)
Disagree	44 (32.1)
Strongly Disagree	29 (21.2)
Generic terms used in the ingredients list (i.e., spices)	
Strongly Agree	60 (43.8)
Agree	62 (45.3)
Neutral	12 (8.8)
Disagree	1 (0.7)
Strongly Disagree	2 (1.5)
E-numbers are provided with no details	
Strongly Agree	39 (28.5)
Agree	58 (42.3)
Neutral	38 (27.7)
Disagree	1 (0.7)
Strongly Disagree	1 (0.7)
Lack of information (e.g., manufacturer’s details)	
Strongly Agree	43 (31.4)
Agree	64 (46.7)
Neutral	23 (16.8)
Disagree	6 (4.4)
Strongly Disagree	1 (0.7)

**Table 8:** Linear regression analysis of food allergen attitude scores with sociodemographic characteristics.

Variable	Simple Regression *β* Coefficient, (95% CI)	*p*-Value	Multiple Regression *β* Coefficient, (95% CI)	*p*-Value
Gender	0.036 (−1.185, 1.258)	0.953		
Age	0.049 (−0.023, 0.121)	0.183		
Marital Status				
Single (ref.)	0			
Married	0.172 (−1.285, 1.63)	0.816		
Nationality (Lebanese)	−0.255 (−1.788, 1.278)	0.743		
Governorate				
Beirut (ref.)	0			
South	0.057 (−1.261, 1.375)	0.932		
North	0.03 (−1.958, 2.018)	0.976		
Bekaa	1.367 (−0.696, 3.429)	0.192		
Mount Lebanon	−0.359 (−1.693, 0.975)	0.595		
Educational Level				
Middle School (ref.)	0			
High School Diploma	−1.583 (−5.187, 2.021)	0.386		
Undergraduate(Bachelor’s Degree)	−0.305 (−3.597, 2.987)	0.855		
Master’s degree	0.725 (−2.592, 4.043)	0.666		
Technical School	5.667 (−0.781, 12.114)	0.084		
Employment Status				
Employed (Full Time) (ref.)	0			
Employed (Part Time)	−0.596 (−2.367, 1.175)	0.507	−0.569 (−2.318, 1.181)	0.521
Seeking employment	−0.068 (−1.521, 1.384)	0.926	−0.081 (−1.516, 1.354)	0.912
Unemployed/Housewife	0.625 (−1.544, 2.794)	0.57	0.336 (−1.825, 2.496)	0.759
Student	−1.202 (−2.387, −0.017)	0.047 *	−1.057 (−2.235,0.122)	0.078
Total Income				
<LBP 1,000,000 (ref.)	0			
LBP 1,000,000–LBP 3,000,000	1.628 (−0.118, 3.374)	0.067		
3,000,000–5,000,000	1.029 (−0.789, 2.846)	0.265		
>5,000,000 LBP	−0.284 (−0.549, 3.032)	0.172		
Previous Experience of a severe reaction	−1.667 (−3.004, −0.33)	*p* < 0.001 *	−1.432 (−2.798, −0.067)	0.04 *

* Estimates shown in bold are those that are statistically significant at *p* < 0.05.

## Data Availability

The data presented in this study are available on request from the corresponding author. The data are not publicly available due to privacy of participants and ethical concerns.
